# Additive Protection by Antioxidant and Apoptosis-Inhibiting Effects on Mosquito Cells with Dengue 2 Virus Infection

**DOI:** 10.1371/journal.pntd.0001613

**Published:** 2012-04-17

**Authors:** Tien-Huang Chen, Yin-Ping Lo, Chao-Fu Yang, Wei-June Chen

**Affiliations:** 1 Graduate Institute of Biomedical Sciences, Chang Gung University, Kwei-San, Tao-Yuan, Taiwan; 2 Department of Public Health and Parasitology, Chang Gung University, Kwei-San, Tao-Yuan, Taiwan; Mahidol University, Thailand

## Abstract

Cytopathic effects (CPEs) in mosquito cells are generally trivial compared to those that occur in mammalian cells, which usually end up undergoing apoptosis during dengue virus (DENV) infection. However, oxidative stress was detected in both types of infected cells. Despite this, the survival of mosquito cells benefits from the upregulation of genes related to antioxidant defense, such as glutathione S transferase (GST). A second defense system, *i.e.*, consisting of antiapoptotic effects, was also shown to play a role in protecting mosquito cells against DENV infection. This system is regulated by an inhibitor of apoptosis (IAP) that is an upstream regulator of caspases-9 and -3. DENV-infected C6/36 cells with double knockdown of GST and the IAP showed a synergistic effect on activation of these two caspases, causing a higher rate of apoptosis (>20%) than those with knockdown of each single gene (∼10%). It seems that the IAP acts as a second line of defense with an additional effect on the survival of mosquito cells with DENV infection. Compared to mammalian cells, residual hydrogen peroxide in DENV-infected C6/36 cells may signal for upregulation of the IAP. This novel finding sheds light on virus/cell interactions and their coevolution that may elucidate how mosquitoes can be a vector of DENV and probably most other arboviruses in nature.

## Author Summary

This study demonstrated an idea that mosquito cells can survive dengue virus (or other arboviruses) infection through antioxidant defense and an additional effect by induction of IAP expression for protection of infection. It makes mosquito eligible to support virus replication efficiently, leading to a goal which is important to explain how mosquitoes can be a vector even when they have been seriously infected by the virus. Our findings opened an avenue for studies on virus/vector co-evolution that benefits for both virus replication and its transmission to humans or susceptible hosts.

## Introduction

The dengue virus (DENV), a flavivirus belonging to the family Flaviviridae, is the etiological agent of dengue fever (DF), dengue hemorrhagic fever (DHF), and dengue shock syndrome (DSS) [1]. The genome of the DENV consists of a single-stranded positive-sense RNA of ∼11 kilobases (kb) long; which possesses an m7G cap at the 5′-end and is non-polyadenylated at its 3′-end [2]. During infection within a host cell, viral RNA directly translates into a single polyprotein that is subsequently cleaved into three structural proteins and seven nonstructural proteins by the combined action of host proteases and the trypsin-like viral NS2B/NS3 serine protease [3]. The DENV and other viruses generally invade a host cell by redirecting cellular processes in order to meet the needs of viral propagation. This, in turn, leads to novel changes in the expressions of various genes [4]–[6].

During infection by the DENV, an unfolded protein response (UPR), or so-called UPR signal cascade, is usually induced in a time-dependent manner [7]; it may be able to cope with endoplasmic reticular (ER) stress in host cells [8]. Moreover, specific serotypes of DENV may modulate the UPR with different selectivities [9]. Generally, the UPR can provide an early defensive mechanism for infected cells to survive ER stress due to viral infection by eliminating misfolded proteins and allowing cells to recover. [10]. In contrast, it might also modify the outcome to benefit viral replication [11]. Despite this, the cell type may also play a role in determining responses to viral infections, leading to differential fates of infected cells [12].

Arboviruses, *e.g.*, DENV, require arthropods as vectors for effective transmission in nature [13], indicating that the viruses can efficiently replicate in both invertebrate and vertebrate cells. It seems that mosquitoes are the mixing tanks of arboviruses. Thus, co-evolution between mosquito cells and viruses is required. However, such viruses apparently cause different outcomes in different cell types [14]. It was reported that apoptosis ultimately occurs in most flavivirus-infected mammalian cells, even though the UPR is activated [15]. Nevertheless, minor cytopathic effects (CPEs) are commonly seen in DENV-infected mosquito cells [16]. In fact, mosquito cells after DENV infection generally overexpress antioxidant genes such as glutathione S transferase (GST) which copes with virus-induced oxidative stress, leading a high survival rate of infected cells [14]. Although this significant reduction in cell death may be mediated by disruption of redox signaling, it is, however, apparently not effective enough to save infected cells according to observations of changes in caspases and apoptosis rates between cells with and without DENV infection [14].

It is conceivable that trivial damage to infected cells is a prerequisite for a mosquito or arthropod to be capable of efficiently transmitting arboviruses in nature. As a result, it is worthwhile determining the occurrence of an antiapoptotic mechanism in mosquito cells in response to DENV infection. It is particularly important to identify what factors may be involved in regulating the occurrence of apoptosis and to investigate how it is induced during infection. We preliminarily identified an inhibitor of apoptosis (IAP) gene that is upregulated in mosquito cells with DENV infection. In this study, we tried to further demonstrate the role of the IAP in DENV-infected mosquito cells and annotate the link between antiapoptotic effects and antioxidant defense which was described previously [14].

## Materials and Methods

### Virus propagation and cell culture

C6/36 cells derived from the mosquito *Aedes albopictus* were used for DENV-2 (New Guinea C strain) propagation; cells were cultured in minimal essential medium (MEM; GIBCO™, Invitrogen, Carlsbad, CA, USA) supplemented with 10% fetal bovine serum (FBS), 2% non-essential amino acids, 2 g/ml Hepes (Sigma, St. Louis, MO, USA), 2.2 g/ml sodium bicarbonate (NaHCO_3_), and 0.4% of an antibiotic-antimycotic (GIBCO™, Invitrogen) in a closed system at 28°C. The propagated virus was titrated as previously described in BHK-21 cells derived from baby hamster kidney [5], which were maintained in MEM containing 10% FBS, 2% non-essential amino acids, 2.2 g/ml NaHCO_3_, and 0.4% of an antibiotic-antimycotic in an atmosphere containing 5% CO_2_ at 37°C.

### Cell infection and RNA extraction

C6/36 cells (∼10^7^ cells/tube) harvested in tubes were centrifuged at 3000 rpm and 4°C for 10 min. The DENV-2 suspension or fresh medium (with mock infection used as the control) was added to the tubes, after removing the medium, at a multiplicity of infection (MOI) of 1 for incubation at 28°C for 1 h with gentle agitation every 15 min. The viral suspension was then removed by centrifugation, and pelleted cells were seeded and incubated at 28°C for 24 h. RNA extraction and reverse-transcription polymerase chain reaction (RT-PCR) procedures were performed following a previous description [4]. Briefly, total RNA was isolated from both mock- and DENV-2-infected C6/36 cells using the Trizol reagent (Invitrogen) following the protocol in the manual provided by the manufacturer, for further experiments.

### Construction of a micro (mi)RNA-based (miR) RNA interference (RNAi) vector for knockdown of the GST gene

The approach followed a protocol to generate a double-stranded (ds)-oligo as a tool of miR RNAi (Invitrogen). The miR RNAi sequence to knock down the GST gene (miR-GST) was generated by annealing the top- and bottom-strand oligos containing the linkers (top: 5′TGCTG….3′; bottom: 5′CCTG….C3′), the mature miR-GST antisense target sequence, the loop sequence, and the sense target sequence (5′cgtgatgtgcctggagggaat3′), to form ds-oligos (top strand: tgctgattccctccaggcacatcacggttttggccactgactgaccgtgatgtctggagggaat; bottom strand: cctgattccctccagacatcacggtcagtcagtggccaaaaccgtgatgtgcctggagggaatc ). Construction of this system was described in our previous report [14]. Transcript changes in the GST gene in cells, either transfected or not, were validated by a real-time RT-PCR using forward (GST-RTF: ACCGAGGATTATGCCAAGATG) and reverse primers (GST-RTR: TCGCACAAATACTGGAGGATG).

### Construction of an miR RNAi vector to knock down the IAP gene

The approach followed a protocol to generate a ds-oligo as a tool of miR RNAi (Invitrogen). The miR RNAi sequence (including 2 fragments of miIAP-1 and miIAP-2) to knock down the IAP gene was generated by annealing the top- and bottom-strand oligos containing the linkers (top: 5′TGCTG…3′; bottom: 5′CCTG…C3′), the mature miR-IAP, the antisense target sequence, the loop sequence, and the sense target sequence (5′TGACGTGTGAGGAAATGATCA3′), to form ds-oligos (top strand: TGCTGTGATCATTTCCTCACACGTCAGTTTTGGCCACTGACTGACTGACGTGTGGAAATGATCA; bottom strand: CCTGTGATCATTTCCACACGTCAGTCAGTCAGTGGCCAAAACTGACGTGTGAGGAAATGATCAC ). Construction of the vector was performed as described previously [14]. Transcript changes in the IAP gene in cells, either transfected or not, were validated by a real-time RT-PCR using the forward (IAP-RTF: ATCGCGAGAAGAGGAGCATC) and reverse primers (IAP-RTR: TGCCATCATCATTTGAGCCA). The primers used for IAP detection were derived from a sequence from the EST dataset previously established by our laboratory. The primer pair used for detection of IAP from BHK-21 cells by real time RT-PCR included the forward (BHIAPRTF: GGAGGCGGTTAGACAGAAG) and reverse primers (BHIAPRTR: GTATAGCACAGGGTCACTCTC).

### Double knockdown of the GST and IAP genes

After constructing the mi-IAP-silenced construct, we transiently transfected the miIAP construct into miGST-C6/36 cells. miGST-C6/36 cells were seeded into 6-well plates 12 h prior to transfection. We prepared the transfection mix reagent containing 100 µl of FBS and antibiotics-free medium, 2 µg of the miIAP construct, and 8 µl FuGENE® HD (Roche, Berlin, Germany), which was then incubated for 15 min at room temperature. After 15 min, miGST-C6/36 cells were washed with antibiotics-free medium without FBS, and such medium was added to the transfection mix reagent to a final volume of 1 ml. We removed the transfection mix reagent from miGST-C6/36 cells, incubated the cells for 5 h, and then replaced the medium with serum-containing medium 24 h after transfection.

### Assays for caspases-9 and 3

Assays for caspases-9 and 3 activated in apoptosis cell followed the protocol of kits provided by the manufacturer (BioVision, Mountain View, CA, USA), which were measured by flow cytometry using caspase inhibitors conjugated to sulfo-rhodamine. Both C6/36 and BHK-21 cells were used for the previously described assay [14].

### Apoptosis rate measured with propidium iodide (PI) nucleic acid staining

The method for measuring cell death followed a previously reported description [14]. In brief, C6/36 cells (∼2×10^6^ cells/tube) with or without transfection of the miR-IAP were collected and infected with DENV-2 at an MOI of 1. At 12, 24, 36, and 48 hpi, cells (both infected and uninfected) were harvested for measurement by flow cytometry.

### Treatment of C6/36 cells with an antioxidant reagent

The monolayer of C6/36 cells inoculated with the viral suspension (at an MOI of 1) was absorbed for 1 h. After the fluid was removed, the antioxidant reagent, N-acetyl-L-cysteine (L-NAC) (Sigma-Aldrich, St. Louis, MO, USA), at a concentration of 10 mM (pH 7.4) adjusted with fresh medium, was added for further culture. Cells were harvested to measure H_2_O_2_, caspases, and apoptosis, as described previously [14].

### Detection of hydrogen peroxide in virus-infected cells

The monolayer of C6/36 cells (with or without transfection of miR-GST) infected with DENV-2 at an MOI of 1 in a Petri dish (10 cm in diameter) was washed with phosphate-buffered saline (PBS; pH 7.3), and then treated with trypsin-EDTA for 5 min. One milliliter of PBS containing 10% FBS was added to the dish, and cells were incubated with 10 µM 2′,7′-dichlorofluorescein (CM-H2DCFDA) (Sigma-Aldrich) at 28°C in the dark for 30 min. Cells were then harvested and subjected to analysis by a fluorescence-activated cell sorter (FACScalibur, Becton Dickinson, Immunofluorometry Systems, Mountain View, CA, USA) with excitation at 535 nm and emission at 610 nm.

### Confocal microscopy for the immunofluorescent assay

An immunofluorescent assay was used to detect localization of double-stranded (ds)RNA or the envelope (E) protein of the DENV-2. Infected and uninfected C6/36 cells were smeared on a cover glass which was then washed with PBS (pH 7.4) three times. Subsequently, cells were fixed in 4% paraformaldehyde for 10 min, washed again with PBS, and then blocked with 1% bovine serum albumin (BSA) in PBS for 1 h at 37°C. Primary polyclonal antibodies (1∶100 in dilution) of anti-dsRNA (J2) (English & Scientific Consulting, Szirak, Hungary) were added to the cover glass and incubated at 37°C for 1 h. The cover glass was subsequently incubated with secondary antibodies (1∶100) conjugated with FITC at 37°C for 1 h after they had been washed with PBS. The cover glass was finally mounted with a mixture of glycerol and PBS (3∶7) and observed under a laser scanning confocal microscope (Zeiss LSM 510, Vertrieb, Germany). Negative controls were incubated with diluents without primary antibodies; otherwise samples were subjected to the same procedures described above. The antibody against protein E of the DENV-2 was kindly provided by Prof. C. L. Kao, Department of Clinical Laboratory Sciences and Medical Biotechnology, National Taiwan University (Taipei, Taiwan).

### Transmission electron microscopy

For electron microscopy, cells seeded on the dish or those scraped off the culture dish (and centrifuged at 4°C and 3000 rpm for 10 min) were immediately fixed with a mixture of 2% (v/v) glutaraldehyde and 2% paraformaldehyde in 0.1 M cacodylate buffer overnight at 4°C. After cells were post-fixed in 1% (w/v) osmium tetroxide in 0.1 M cacodylate buffer for 2 h at room temperature, they were washed with 0.2 M cacodylate buffer three times. Again, cells were washed with 0.2 M cacodylate buffer three times and then dehydrated through an ascending graded series of ethanol. Cells were finally embedded in Spurr's resin (Electron Microscopy Science, Hatfield, PA, USA) and polymerized at 70°C for 72 h. The trimmed blocks were sectioned with an ultramicrotome (Reichert Ultracut R, Leica, Vienna, Austria). Ultrathin sections were sequentially stained with saturated uranyl acetate in 50% ethanol and 0.08% lead citrate. Selected images were observed and photographed under an electron microscope (JEOL JEM-1230, Tokyo, Japan) at 100 kV.

### Statistical analysis

Comparisons between two means were analyzed by Student's *t*-test at a significance level of 5%.

## Results

### Activities of caspases in host cells with DENV infection

**Figure 1:**
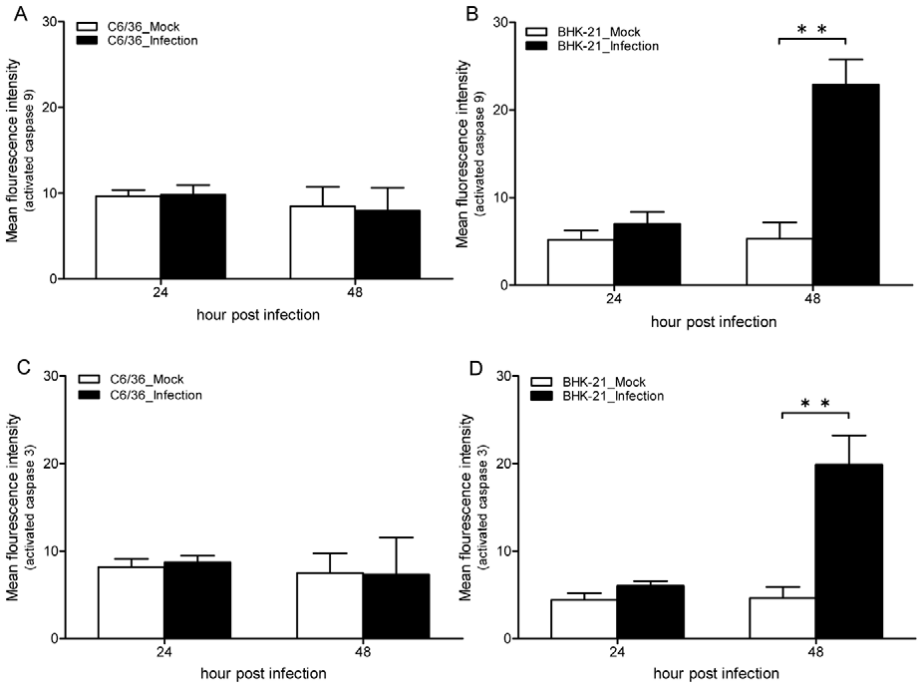
Changes in activities of activated caspases-9 and -3 in C6/36 and BHK-21 cells infected with the dengue 2 virus at a multiplicity of infection (MOI) of 1 for 24 and 48 h. Caspase-9 did not significantly change in C6/36 cells at 24 h post-infection (hpi) (A) while it had increased 3.3-fold by 48 hpi in BHK-21 cells with viral infection (B). Similarly, caspase-3 insignificantly changed in C6/36 cells (C), while it had increased 3.4-fold by 48 hpi in infected BHK-21 cells (D).

In this study, we assessed changes in caspases and their effects on apoptosis occurring among DENV-infected mosquito cells. Our results revealed that no evident change in activated caspase-9 occurred in C6/36 cells even when they had been infected by the virus for 48 h (Fig. 1A), while it had increased 3.3-fold at 48 h hpi in infected BHK-21 cells which usually ended up dead (Fig. 1B). Similarly, activated caspase-3 remained at a low level (<10 mean fluorescent intensity; mfi) in both C6/36 and BHK-21 cells even after they had been infected with DENV for 24 h (Fig. 1C). However, it had significantly increased to 3.4-fold in BHK-21 cells, but not C6/36 cells, at 48 hpi (Fig. 1D).

### Inhibitor of apoptosis (IAP) expression and its effect on apoptosis of infected cells

**Figure 2:**
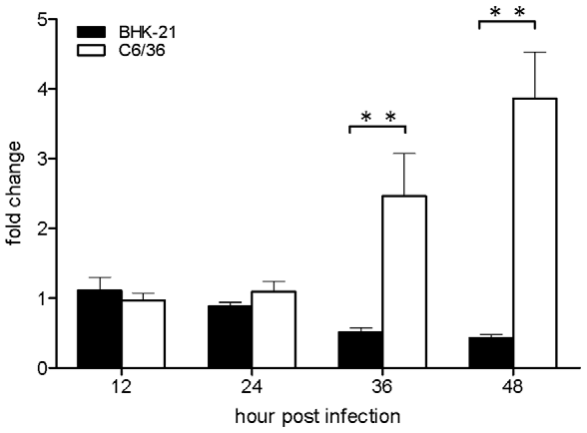
Expressions of the inhibitor of apoptosis (IAP) in C6/36 and BHK-21 cells detected at 12∼48 h post-infection (hpi). Expression of the IAP had slightly decreased by 50% and 60% reductions at 36 and 48 hpi, respectively, in infected BHK-21 cells. In contrast, it had gradually increased by 2.4- and 3.8-fold at 36 and 48 hpi, respectively, in C6/36 cells.

**Figure 3:**
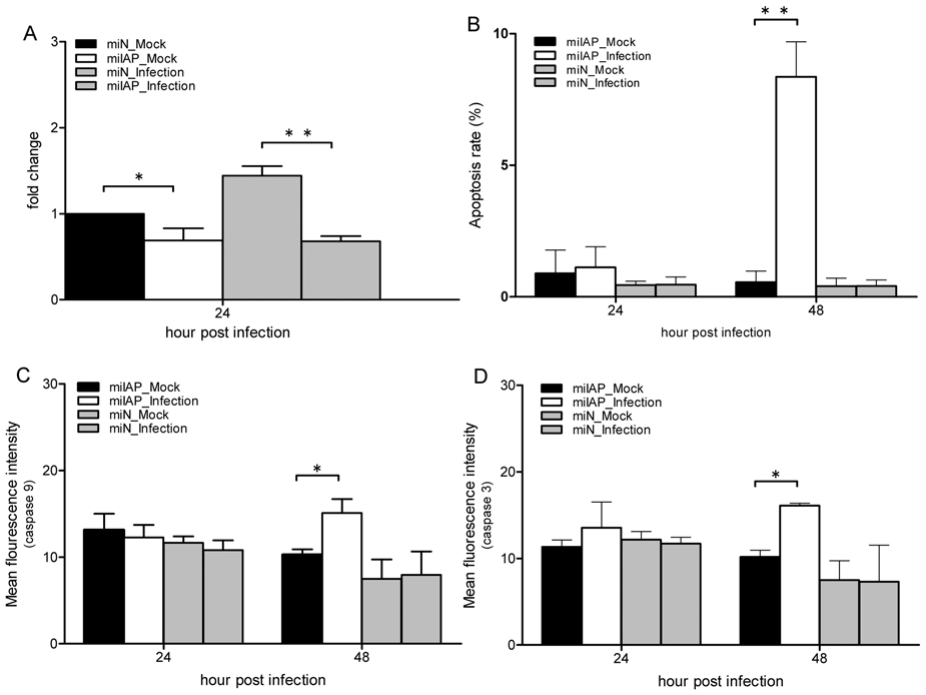
Expression of the inhibitor of apoptosis (IAP) in C6/36 cells in which the IAP had been knocked down. The results showed about 50% efficiency of knockdown although it had increased 1.9-fold by 24 h post-infection (hpi) (A). The apoptosis rate significantly increased by 8.3% in C6/36 cells with knockdown of the IAP after infection with the dengue 2 virus (DENV-2) for 48 h, while no difference was apparent at 24 hpi. The apoptosis rates in other groups were all <1.2% (B). Activities of caspases-9 (C) and -3 (D) also had significantly increased only at 48 hpi in DENV-2-infected C6/36 cells with knockdown of the IAP. * *p*<0.05; ** *p*<0.01; miIAP: cells with IAP knockdown; mN: control cells without IAP knockdown.

In DENV-infected C6/36 cells, the IAP gene was significantly 2.4-fold upregulated at 36 hpi, and had reached 3.8-fold by 48 hpi; however, it had decreased to 50% and 60% by 36 and 48 hpi, respectively, in infected BHK-21 cells (Fig. 2). This suggests that the IAP plays a role in this process. It implies that an antiapoptotic effect through the IAP serves as a second defense system, protecting cells from virus-induced apoptosis in C6/36 cells. In this study, we also applied an miRNA-based silencing technique to knock down the IAP in C6/36 cells, and obtained a 50% knockdown efficiency (Fig. 3A). Through this knockdown system, the apoptosis rate was evaluated, and showed an increase of 8.3% in those cells at 48 hpi (Fig. 3B). In the meantime, elevated (1.46-fold) caspase-9 activity appeared in IAP-knockdown and DENV-infected C6/36 cells at 48 hpi (Fig. 3C), while caspase-3 showed an increase of 1.54-fold at the same time after infection (Fig. 3D).

### Additional effects of the IAP and GST on the survival of C6/36 cells

**Figure 4:**
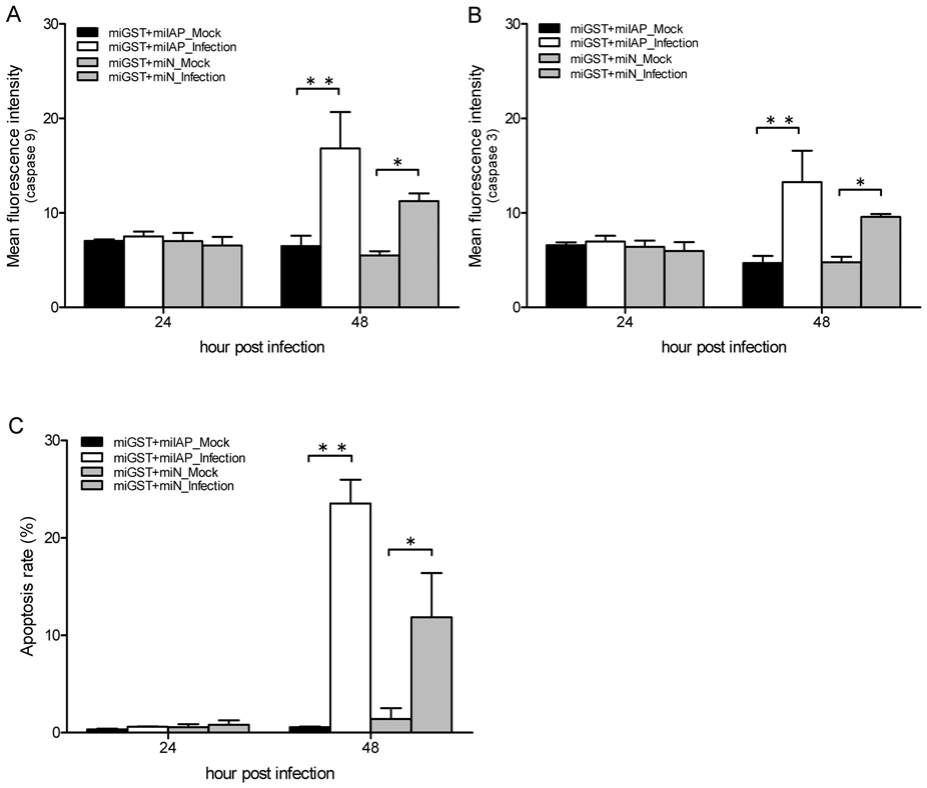
Responses of dengue 2 virus (DENV-2)-infected C6/36 cells with double knockdown of glutathione S transferase (GST) and the inhibitor of apoptosis (IAP). Activities of caspases-9 (A) and -3 (B), and the apoptosis rate (C) had significantly increased at 24 and 48 h post-infection (hpi) in infected C6/36 cells with double knockdown of the IAP and GST. All results revealed significant differences in caspases and apoptosis rates between singly and doubly knocked-down cells. * *p*<0.05; ** *p*<0.01.

It was observed that caspases are significantly activated in BHK-21 cells infected with DENV, producing a high proportion of cell death through apoptosis. In contrast, C6/36 cells with DENV infection upregulated IAP expression which suppressed the activities of caspases-9 and -3, leading to an antiapoptotic effect in C6/36 cells as shown above. We thus further doubly knocked down C6/36 cells to reduce expressions of the IAP and GST. Cells with DENV infection revealed that caspase-9 had increased 2.6-fold at 48 hpi, although it was at a low level at 24 hpi (Fig. 4A). The pattern of caspase-3 activation was similar to that of caspase-9; its activity had increased 2.83-fold by 48 hpi (Fig. 4B). These results showed a significant difference in caspase activities between singly and doubly knocked-down cells. The apoptosis rate had increased to 23.52% for doubly knocked-down C6/36 cells with DENV infection by 48 h, which significantly differed from that of cells with single knockdown of the GST gene (Fig. 4C). These results revealed there are two separate lines of defense involved in reducing apoptosis in mosquito cells with DEVN infection: one operated by GST and the other by the IAP.

### Imaging evidence of DENV replication in C6/36 cells

**Figure 5:**
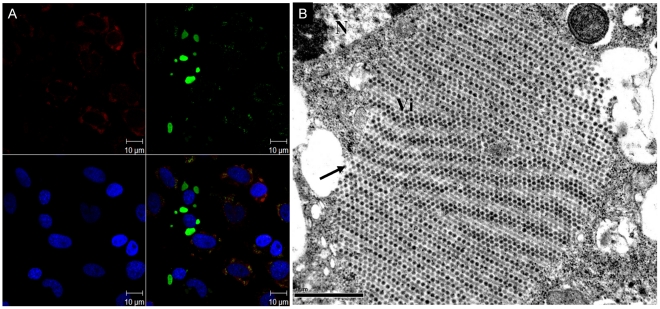
Imaging evidence of dengue 2 virus (DENV-2) replication within C6/36 cells. (A) Both double-stranded (ds)RNA (green) and the viral E (envelope) protein (red) frequently appeared in C6/36 cells after infection with DENV-2 at 12 and 24 hpi, respectively. This indicates that RNA replication and protein synthesis of the virus were active at these time points. That shown blue represented nuclei stained with DAPI. A merged image was shown at the right bottom of this figure. (B) A great number of virions (Vi) which formed as a crystalline array appeared in membrane-bound vacuoles (arrow) in C6/36 cells infected by the virus for 24 h. N, nucleus, Scare bar = 1 µm.

In C6/36 cells infected with DENV at an MOI of 1, dsRNA, representing active viral RNA replication, and E proteins, representing the synthesis of viral proteins, were frequently observed at 12 hpi (Fig. 5A). In the meantime, a great number of progeny virions that formed as a crystalline array within membrane-bound vacuoles widely appeared, mostly at 24 hpi, in the cytoplasm (Fig. 5B). It was noted that C6/36 cells were morphologically intact even though thousands of virions have formed in those cells.

### Production of H_2_O_2_ and its dynamics in DENV-infected C6/36 cells

**Figure 6:**
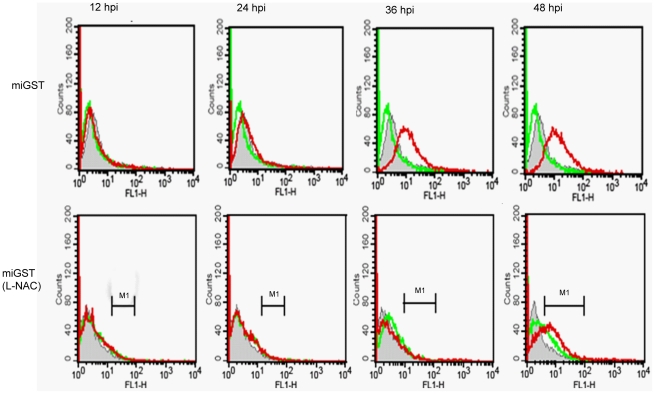
Intracellular H_2_O_2_ measured through CM-H2DCFDA staining followed by flow cytometry. (A) H_2_O_2_ was elevated at 36∼48 h post-infection (hpi) in dengue 2 virus (DENV-2)-infected BHK-21 and C6/36 cells with knockdown of glutathione S transferase (GST). However, it had rather slightly increased by 36 hpi, although it was still higher at 48 hpi.

The present study measured H_2_O_2_, by CM-H2DCFDA staining, produced in DENV-infected BHK-21 and C6/36 cells, which showed a significant difference between the two cell types. The former usually presented vigorous H_2_O_2_ elevation, basically beginning at 36 hpi and remaining through 48 hpi, while the latter revealed a slight increase at 36 hpi which had further increased by 48 hpi. However, C6/36 cells with GST knockdown presented a pattern of H_2_O_2_ production similar to that of BHK-21 cells (Fig. 6). This indicates that H_2_O_2_ increased much later and more slightly in mosquito cells in response to DENV infection, suggesting that a stable environment remained in C6/36 cells particularly in the early stage of infection.

### Regulation of IAP expression by the antioxidant status in infected C6/36 cells

**Figure 7:**
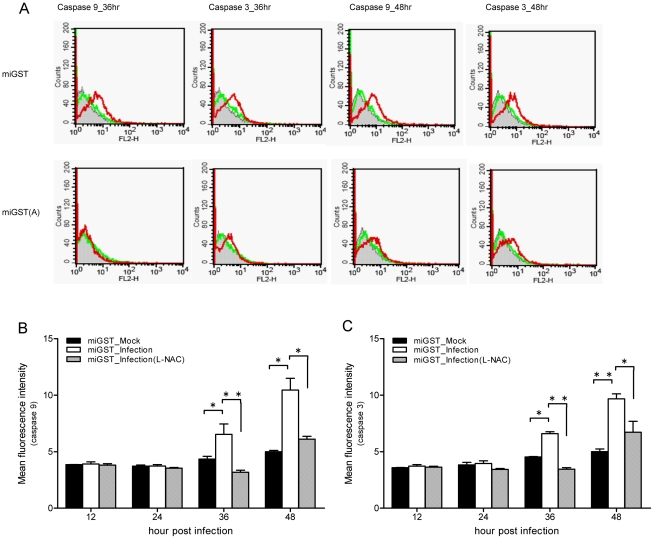
Activity changes of caspases in miGST-C6/36 cells with or without L-NAC treatment. (A) Both caspases-9 and -3 were activated at 36∼48 h post-infection (hpi), while only minimal activation was shown at 48 hpi in cells treated with L-NAC. (B) Caspase-9 had significantly increased by 36 hpi in glutathione S transferase (GST)-knockdown C6/36 cells after infection with the dengue virus (DENV), but decreased after treatment with L-NAC. (C) Caspase-3 had also significantly increased by 36 hpi in GST-knockdown C6/36 cells after infection with the DENV, while it decreased after treatment with L-NAC.

**Figure 8:**
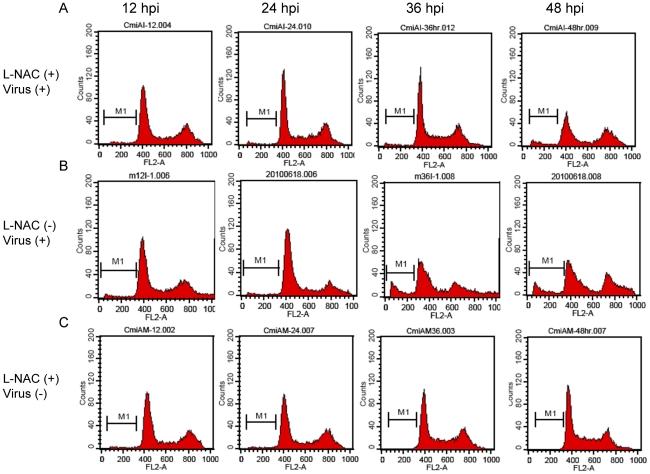
Effects of L-NAC on the death of glutathione S transferase (GST)-knockdown C6/36 cells with dengue 2 virus (DENV-2) infection. (A) When GST-knockdown C6/36 cells were treated with L-NAC, the apoptosis rate was no higher than 5.69% (at 48 h post-infection; hpi) in infected cells. (B) In contrast, DENV-2 infection without treatment resulted in 10.45% and 11.08% apoptosis rates at 36 and 48 hpi, respectively. (C) Apoptotic effect of L-NAC was not evident on uninfected cells.

**Figure 9:**
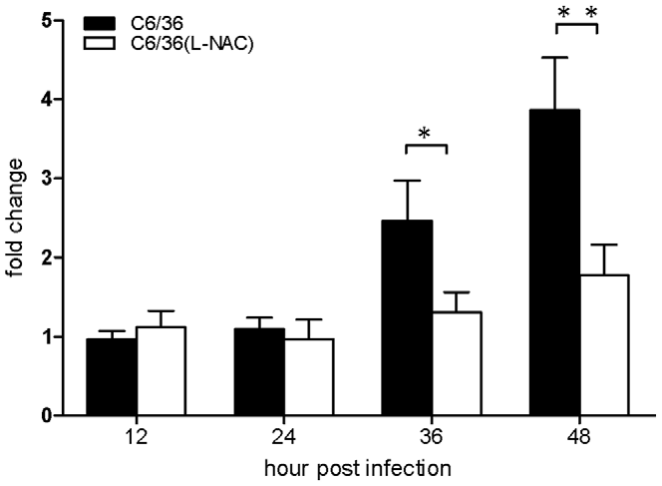
Expression of the inhibitor of apoptosis (IAP) in C6/36 cells was affected by treatment with the antioxidant, L-NAC. The result showed significant suppression at 36 and 48 h post-infection with the dengue 2 virus.

The sulfhydryl antioxidant, L-NAC (10 mM), was used to treat C6/36 cells with and those without GST-knockdown (miGST) in order to compensate for the antioxidant ability within cells. In GST-knockdown C6/36 cells, both caspases-9 and -3 were activated at 36∼48 hpi, while only minimal activation was apparent at 48 hpi in cells treated with L-NAC (Fig. 7A). Quantitative results showed that caspase-9 had significantly increased by 36 hpi in GST-knockdown C6/36 cells after infection; however, it decreased after treatment with L-NAC (Fig. 7B). Production of caspase-3 also showed a similar trend to that of caspase-9 (Fig. 7C). Looking at the apoptosis rate in GST-knockdown C6/36 cells, L-NAC treatment obviously alleviated the apoptosis rate to a relatively low level (5.69% at 48 hpi) (Fig. 8A). In contrast, untreated cells had a higher apoptosis rate at 36 hpi (10.45%) which had further increased to 11.08% by 48 hpi (Fig. 8B). The apoptotic effect of L-NAC treatment on uninfected cells has been demonstrated to be extremely low (Fig. 8C). Our observations showed that the antioxidant status is undoubtedly associated with elevated expression of the IAP which ultimately protects DENV-infected C6/36 cells. Treatment with L-NAC to strengthen the antioxidant ability, presumably by altering the antioxidant status, actually alleviated IAP expression in C6/36 cells (Fig. 9). In C6/36 cells without L-NAC treatment, induction of the IAP usually increased in response to DENV infection, particularly at 36 and 48 hpi (Fig. 9).

## Discussion

Many flaviviruses have been known to evolve specific tactics, resulting in evasion of the innate and adaptive immune responses [17]; which usually occur among mammalian hosts in response to infection of the viruses [18], [19]. However, it seems like mosquitoes preferentially select innate responses due to lack of an adaptive immunity [20]. For instance, Toll, Imd (immune deficiency), and JAK-STAT pathways are frequently implemented by insects including mosquitoes, those pathways usually play a role in limiting viral replication within host cells [21]. According to reported, flaviviriral infections of mosquito cells can also be modulated by the mosquito's RNA interference pathway [22].

In general, mosquito cells are unable to use a potent cellular response to eliminate viral infected cells as mammalian cells. It suggests that a compromising between host cells and viral replication may be the best choice for mosquito survival. In turn, it has recently been reported that mosquito cells may utilize antioxidant defense as one mechanism to protect themselves [14]. In the meantime, inducing or inhibiting apoptosis may also be involved in affecting arbovirus replication in mosquito cells [23].

We previously demonstrated that mitochondria are involved in the caspase cascade and resulting apoptosis in host cells with DENV infection [14], likely via the so-called mitochondrial or intrinsic pathway. Since apoptosis is inevitable in DENV-infected BHK-21 cells, activation of both caspase-9 and -3 is apparently essential in those cells. Theoretically, activation of caspase-9 disrupts mitochondrial diffusion limits and leads to the further release of cytochrome (Cyt) *c* [24]. In turn, Cyt *c*-dependent formation of an Apaf-1/caspase-9 complex activates caspase-3 and eventually initiates an apoptotic protease cascade [25]. Since C6/36 cells usually survive DENV infection, it is thought that there must be a negative regulator or suppressor of apoptosis that protects C6/36 cells infected by the DENV.

Apoptosis is important for a variety of life phenomena [26]; it can also be a cellular response to viral infections [27]. Caspases are central components of the machinery responsible for apoptosis and/or programmed death of cells [28]; they belong to a group of enzymes known as cysteine proteases [29]. It is now known that apoptosis is induced via either activation of death receptors (the extrinsic pathway) or mitochondria (the intrinsic pathway). In most cases, both pathways converge to induce activation of caspases which are cleaved to form the active forms before apoptosis occurs [30]. Induction of apoptosis via death receptors typically results in activation of an initiator caspase such as caspase-8 or -10, which then activate other caspases in a cascade, eventually leading to activation of effector caspases, such as caspases-3 and -6 [31]. On the other hand, the mitochondrial pathway of apoptosis begins with a change in the permeability of the mitochondrial outer membrane [32]. Generally, activation of caspase-9 (the initial caspase) followed by caspase-3 (the effector caspase) is mediated by the formation of an apoptosome, leading to the occurrence of apoptosis [33].

Caspases are normally suppressed by IAPs that bind to and inhibit active caspases [34]. IAPs are a family of proteins originally identified in baculoviruses [35], which play roles in protecting infected cells from death [36]. In turn, IAPs act as regulators to inhibit activation of caspases and thus dysregulate cell death pathways [37]. The IAP identified from the mosquito *Ae. albopictus* is composed of 402 amino acids and contains two baculoviral IAP repeat (BIR) domains and a RING-finger domain at its carboxyl terminus [38]. Significant upregulation of the IAP gene in DENV-infected C6/36 cells suggests that IAPs play roles in protecting cells from virus-induced apoptosis [38], [39]. It further implies that an antiapoptotic effect through IAPs serves as a second defense system, leading to the absence of apoptosis in C6/36 cells during DENV infection [36]. We applied an miRNA-based silencing technique to knock down the IAP in C6/36 cells in this study, and showing increased apoptosis and elevated activities of caspases-9 and -3. These results completely coincided with the pathway of intrinsic apoptosis, and suggested that loss of the IAP makes mosquito cells highly sensitive to virus-induced oxidative stress, representing an important regulator of inhibition in nature [40].

We further doubly knocked down C6/36 cells to reduce the expressions of the IAP and GST and show significant differences in caspase activities between singly and doubly knocked-down cells. This suggests that mosquito cells have two strategies, one operated by GST and the other by the IAP, to cope with stresses by inducing genes associated with defense against stress. However, this greatly differs from antiviral strategies in mammalian cells [41], a high proportion of which end up undergoing apoptosis. Multiple defense responses through activation of various defense-related genes were observed in Cucumber mosaic virus (CMV)-infected tomato plants [42]. In this study, we further confirmed an additional effect implemented by the IAP and GST, leading to enhanced protection of C6/36 cells against DENV infection.

We have repeatedly observed that a high survival rate of mosquito cells with DENV infection. Although the antioxidant defense by GST partially contributes to the ability to protect cells, there is supposedly a second defense system to enhance or synergize protection of mosquito cells from DENV infection. It is believed that the antiapoptotic pathway of the IAP plays such a role in elevating protection of infected mosquito cells. Since H_2_O_2_ can act as a redox signal at a lower level within cells [43], it was demonstrated to modulate downstream signaling events such as calcium mobilization, protein phosphorylation, and gene expression [44]. As the result shown above, only a slight amount of H_2_O_2_ was produced in mosquito cells with DENV infection. In the present study, the antioxidant, L-NAC, was used to treat C6/36 cells with GST-knockdown (miGST) in order to neutralize free radicals and consequently protect cells from oxidative stress [45]. Results revealed that antioxidant-deficient C6/36 cells could effectively stimulate IAP expression through recovery by L-NAC treatment. It seems that the antioxidant status can regulate expression of the IAP and the resultant cell fate. In other word, H_2_O_2_ may adjust itself to act as a key player to overcome stress-mediated apoptosis [46], inducing the IAP which serves as a second defense pathway to enhance protection of C6/36 cells from DENV infection. Although arbovirus infection of mosquito cells may trigger cell death, it occurs only when virus replication exceeds a threshold level [21]. This reflects that conventional innate defense of mosquitoes may delay virus replication, leading to a prosperous status of virus growth at the later stage within infected cells.

Overall, this study provided important evidences that mosquito cells can survive DENV infection via an antioxidant defense which is followed by an antiapoptotic effect. These two defense systems are linked by an appropriate dose of residual H_2_O_2_ which was reported to be critical for apoptosis inhibition induced by UV irradiation [47]. This exquisite defense network promotes cell survival of infected mosquitoes; trivial damage to infected cells may be a prerequisite for mosquitoes and arthropods serving as efficient transmitters of arboviruses in nature.
